# Hybrid energy storage configuration method for wind power microgrid based on EMD decomposition and two-stage robust approach

**DOI:** 10.1038/s41598-024-53101-4

**Published:** 2024-02-01

**Authors:** Xiuyu Yang, Xiaoyu Ye, Zhongzheng Li, Xiaobin Wang, Xinfu Song, Mengke Liao, Xueyuan Liu, Qi Guo

**Affiliations:** 1 School of Electrical Engineering, Northeast Electric Power University, JiLin, 132012 China; 2 State Grid Xinjiang Electric Power Co., Ltd. Economic Technology Research Institute, Urumqi, 830002 China; 3 State Grid Hebi Power Supply Company, HeBi, 458000 China

**Keywords:** Electrical and electronic engineering, Energy infrastructure

## Abstract

Data centers are usually characterized by high energy loads, which raises increasing sustainability concerns in both academic and daily usage. To mitigate the uncertainty and high volatility of distributed wind energy generation, this paper proposes a hybrid energy storage allocation strategy by means of the Empirical Mode Decomposition (EMD) technique and the two-stage robust method. First, this paper conducts the evolution analyses for the over- and under-evaluated uncertainty of wind power fluctuation under different time scales. Second, we employ the EMD technique to configure a high-frequency flywheel energy storage device, realizing the wind power transformation from large fluctuations to small fluctuations and the convergence of the wind power fluctuation curves in minute- and hour levels. Finally, based on the hour-level wind energy stable power curves, we carry out two-stage robust planning for the equipment capacity of low-frequency cold storage tanks and lithium bromide chillers. The case study on a data center microgrid in northeastern China confirms the effectiveness of our proposed strategy.

## Introduction

As the Internet gradually integrates into people's lives, the data throughput in various industries has also reached an explosive period. In order to better handle the massive flow of data, the global scale of data centers is continuously increasing. However, with the expansion of data center sizes, their power consumption is also rising year by year. According to statistics, the electricity consumption of global data centers has already accounted for 1.3% of the world's total power supply^1^. In 2020, the proportion of power consumption in China's data centers to the national electricity consumption reached 2.7%^2^. Data centers are generally divided into two types: independent data centers, characterized by small scale and no data transmission with other centers, and internet data centers, typically larger in scale and involved in data transmission, resulting in significant power consumption. According to statistics, the power consumption of Microsoft's data center in Washington has reached 48 MW, equivalent to the power consumption of about 40,000 households^3^. To reduce the high electricity costs of data centers, current operators tend to make greater use of renewable energy. For the reliability of their power supply, operators usually deploy flexible resources such as energy storage and gas turbines to facilitate the integration of wind power. Under the influence of various efforts by operators, data centers are gradually evolving into microgrid systems.

Meanwhile, reducing operational costs while ensuring the stable operation of data centers has become a current research hotspot. Research on data centers mainly focuses on two aspects: optimizing and improving data center operational strategies^4–10^ and selecting and sizing equipment within data centers^11–15^. As data centers gradually transition into microgrids, the smoothing of wind power fluctuations becomes crucial for enhancing the stability of data center operations. However, there is limited research on the internal smoothing of wind power within data centers. Establishing a reasonable strategy for integrating wind power into data centers is currently a major challenge. As a microgrid, a data center exhibits significant differences in wind power frequency fluctuations compared to conventional large power grids^16^. Due to the volatility of wind power, the frequency and peaks vary across different time scales, with minute-level fluctuations far exceeding those at the hourly level. Therefore, large-scale wind farms often employ day-ahead scheduling. Microgrid scheduling strategies, ensuring power supply reliability, include day-ahead scheduling, intra-day scheduling, real-time scheduling^17^, hybrid approaches^18,19^, etc. Data centers, as a novel microgrid system, have a wind power scale much smaller than typical microgrid systems. Long-time scale wind power scheduling strategies are inadequate to address the high-frequency nature of data center wind power systems. Reducing the scale of wind power significantly increases the difficulty of smoothing wind power fluctuations within data centers.

Furthermore, the uncertainty of wind power further increases the difficulty of microgrid scheduling. Therefore, current scholars propose multi-scale microgrid scheduling strategies. In^20^, a multi-time scale optimization and management method for a park's comprehensive energy system based on double-layer deep reinforcement learning is presented. This method constructs upper and lower deep deterministic management models, incorporating comprehensive energy sources, including gas, heat, and electricity. In Ref.^21^, a three-stage economic dispatch model for source-load coordination, covering day-ahead, intra-day, and real-time stages, is proposed. The model optimizes the allocation plan for system load and spinning reserve, addressing issues related to load shedding and wind power curtailment. Reference^22^ introduces an optimization method for energy storage capacity considering the randomness of source load and the uncertainty of forecasted output deviations in a microgrid system at multiple time scales. This method establishes the system's energy balance relationship and a robust economic coordination indicator. However, for multi-time scale scheduling optimization, the significant difference between minute-level and hourly-level power fluctuations can lead to imbalances in system flexibility when applying originally robust strategies designed for hourly operations at the minute level. For instance, changing the time scale to the minute level increases the uncertainty range of predicted power deviations, rendering minute-level scheduling results less informative for day-ahead and real-time scheduling.

This paper proposes Hybrid Energy Storage Configuration Method for Wind Power Microgrid Based on EMD Decomposition and Two-Stage Robust Approach, addressing multi-timescale planning problems. The chosen hybrid energy storage solutions include flywheel energy storage, lithium bromide absorption chiller, and ice storage device. The flywheel energy storage is utilized to smooth the high-frequency components of wind power obtained through EMD decomposition. For the decomposed low-frequency wind power, it is extrapolated to an hourly timescale for subsequent two-stage robust optimization in the data center. This approach overcomes scheduling challenges across multiple timescales, enhancing the flexibility of scheduling methods. By employing the lithium bromide chiller and ice storage system, the cooling load of the data center is increased, working in conjunction with the water cooling system to stabilize the heat load of the data center. This, in turn, improves the economic efficiency of data center operations. Firstly, the initial architecture, transformation plans, and planning process of the research object, the "wind power-included data center microgrid," are elucidated. Secondly, an analysis of the mathematical evolution patterns of wind power uncertainty within the microgrid is conducted, focusing on aggregating uncertainty from the minute level to the hourly level. Subsequently, the introduction of flywheel energy storage devices is employed to transform the minute-scale strong fluctuations of wind power into hour-scale weak fluctuations. Through Empirical Mode Decomposition (EMD), minute-scale high-frequency wind power fluctuations are identified, and an optimal capacity planning strategy for the flywheel energy storage is devised using a variable baseline capacity allocation approach. This aims to absorb the high-frequency wind power components identified through EMD, smoothing the overall output power of both wind power and the flywheel energy storage device. This approach reduces the minute-scale wind power fluctuations, addressing the contradiction in traditional two-stage robust planning where the guidance on hour-scale and minute-scale varies. Finally, the low-frequency components decomposed by EMD and the near-linear negative power components generated after smoothing by the flywheel energy storage are combined as the baseline power of the robust planning wind power box uncertainty set. The two-stage robust planning model of the data center microgrid is solved using the Column and Constraint Generation Algorithm (C&CG), achieving planning results that balance system operating costs and investment costs. Based on this, optimal capacity configurations for bromine lithium absorption chillers and cold storage tanks are determined. Case studies are conducted to verify the effectiveness and optimality of the proposed strategy.

## Methods

### Overview of the basic planning scheme

**Figure 1:**
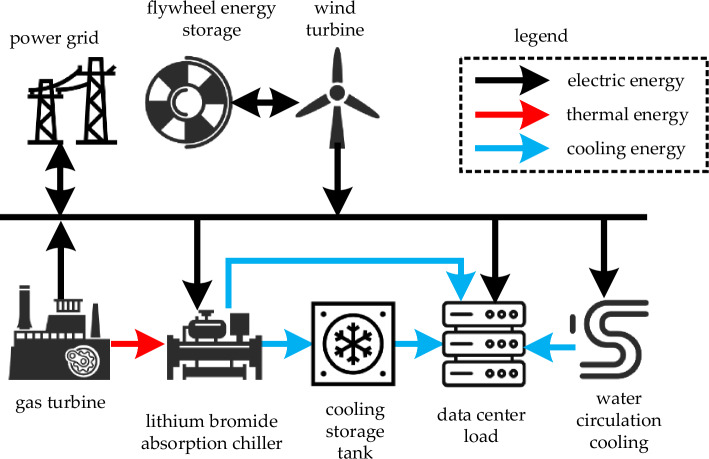
Architecture of a transformed data center microgrid with wind power.

All analyses of this paper are based on the planning Scheme for a Microgrid Data Center with Wind Power, which is illustrated in Fig. 1. The initial architecture of the data center microgrid includes a grid power supply, distributed renewable energy units such as wind power, gas turbines, data center loads, and a water circulation cooling system.

**Figure 2:**
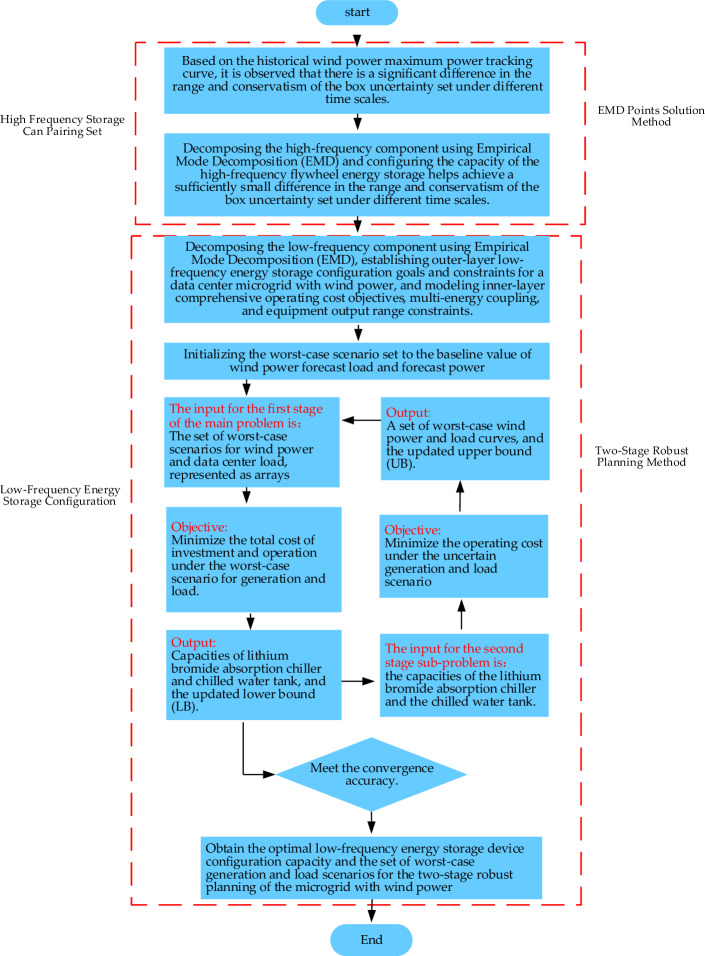
Wind microgrid hybrid energy storage allocation strategy process based on EMD decomposition and two-stage robust method.

As shown in Fig. 1, the renovation plan involves the installation of a flywheel energy storage system to dampen the high-frequency fluctuations in wind power, promoting the overall smoothing of output power from both wind power and the flywheel energy storage system, thus enhancing system stability. Additionally, the plan includes the installation of a bromine lithium absorption chiller to utilize the waste heat from a gas turbine for cooling purposes. Furthermore, a cold storage tank is configured to enable the storage and time-shifting of cooling power, enhancing system economic efficiency. To provide a clearer and more intuitive explanation of the logical sequence of the wind power microgrid hybrid energy storage configuration strategy based on Empirical Mode Decomposition (EMD) and a two-stage robust planning method, a flowchart is depicted in Fig. 2 below.

### Wind power microgrid and empirical mode decomposition

**Figure 3:**
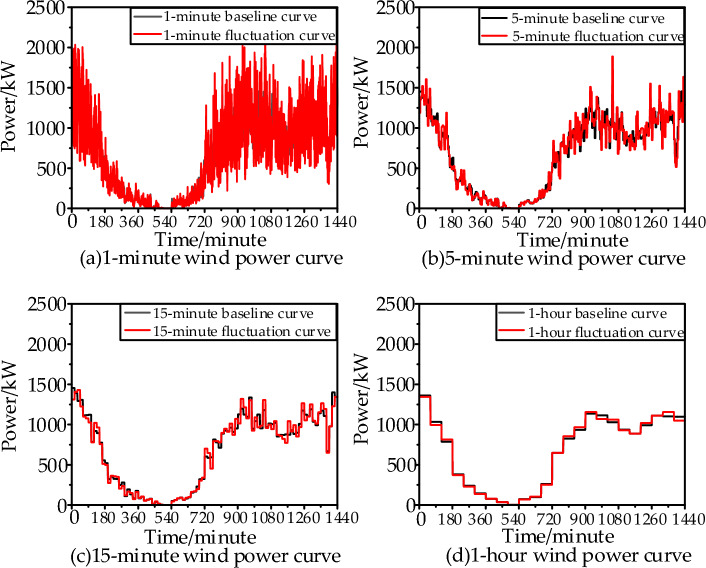
Wind power fluctuation curves at each time scale.

When using the box uncertainty set to evaluate the volatility of wind power, there are mainly two parameters: the fluctuation range and conservatism. Taking the example of random and uniform fluctuations of wind power within a 50% range around the baseline at a 1-min time scale, the changes in wind power fluctuations at 5, 15, and 60-min time scales are shown in Fig. 3.

From Fig. 3, it can be observed that during the evolution from the short time scale of minutes to the long time scale of hours, the wind power curve becomes increasingly smooth.

1 $$\frac{{E_{t}^{Windm} - \widehat{E}_{t}^{Windm} }}{{E_{t}^{Windm} }} \in \left[ - D_{\max }^{EWindm} ,D_{\max }^{EWindm} \right]$$

In the equation, $E_{t}^{Windm}$ represents the predicted maximum wind power, $\hat{E}_{t}^{Windm}$ represents the stochastic wind power, and $D_{\max }^{EWindm}$ is the wind power fluctuation range.

2 $$\sum\nolimits_{t = 1}^{T} {\frac{{\left| {E_{t}^{Windm} - \widehat{E}_{t}^{Windm} } \right|}}{{D_{\max }^{EWindm} \cdot E_{t}^{Windm} }}} = \frac{\Gamma }{T}$$

In the equation, *T* is the total number of hours in a day at a certain time scale, and Γ is the conservatism parameter of the wind power fluctuation box model. Then, for quantitative analysis of the figure above, characteristic parameters of the wind power box fluctuation model for different time scales can be calculated using Eqs. (1), (2), as shown in Table 1 below:

**Table 1:** Comparison of characteristic parameters of wind power box fluctuation models at multiple time scales.

Fluctuation characteristic parameters	1 min	5 min	15 min	60 min
Maximum fluctuation range	51%	39%	26%	8%
Proportion of total fluctuation	25%	11%	6%	3%
Volatility conservation	365/1440	152/1440	87/1440	38/1440

From the example results in the above Table 1, the following conclusions can be drawn:

1. The wind power fluctuation power at short time scales tends to decrease when averaged over longer time scales due to the fusion of positive and negative fluctuations.
2. The deviation between the adverse fluctuation curve and the baseline prediction curve is smaller at longer time scales, while it is larger at shorter time scales. Therefore, making dispatch decisions based on wind power at longer time scales results in more reliable and referenceable decision outcomes. Considering the wind power scale in the load center, it is necessary to overcome the dispatch challenges at different time scales. Consequently, addressing how to improve wind power consistency at various time scales becomes a key issue that needs urgent resolution.

In the following sections, we will address this contradiction by first using EMD decomposition based on high and low frequencies for the capacity configuration of flywheel energy storage, introducing practical controllable energy storage smoothing hardware devices for the mathematical model transformation from 1-min high-frequency large fluctuations to hourly low-frequency small fluctuations. Furthermore, based on the conclusion that "the deviation between the adverse fluctuation curve and the baseline prediction curve is smaller at longer time scales" and "decision results at longer time scales are more reliable and referenceable," a two-stage robust planning is conducted at the 1-h long time scale to enhance the system's robust economic performance.

Utilizing Empirical Mode Decomposition (EMD) for processing the original wind power signal, various intrinsic mode functions (IMFs) are obtained. Due to the unnecessary workload associated with individually processing each IMF component, and considering the wind power integration fluctuation constraints, the signal is reconstructed into low-frequency and high-frequency components. The low-frequency component is utilized for the subsequent two-stage robust economic calculations mentioned later, while the high-frequency component is smoothed using flywheel energy storage, as shown in Eq. (3).

3 $$E^{Windm} = E_{low}^{Windm} + E_{hig}^{Windm} + R^{Windm}$$

where *E*^*windm*^ represents the initial wind power signal, $E_{low}^{Windm}$ denotes the low-frequency component obtained through EMD decomposition, $E_{hig}^{Windm}$ represents the high-frequency component extracted through EMD decomposition, and *R*^*windm*^ represents the residual quantity.

EMD relies on the time-scale characteristics of the data for signal decomposition, exhibiting significant advantages in handling non-stationary data. The steps for high and low-frequency decomposition of non-stationary and nonlinear wind power using the EMD method are as follows:

Identify all extreme points of the wind power signal $E_{i}^{Windm}$ at the 1-min time scale, where $E_{i}^{Windm}$ refers to the remaining power curve after i decompositions.

Use cubic spline interpolation to fit upper and lower envelope lines, $E_{i,\min }^{Wind}$ and $E_{i,\max }^{Wind}$, to the extreme points of the $E_{i}^{Windm}$ curve. The average of the envelope lines is called the Intrinsic Mode Function (IMF), denoted as $IMF_{i}^{Windm}$ Removing it enables the update of wind power high and low-frequency decomposition.

4 $$\left\{ \begin{gathered} E_{i + 1}^{Windm} = E_{i}^{Windm} - IMF_{i}^{Windm} \hfill \\ IMF_{i}^{Windm} = \frac{{E_{i,\max }^{Windm} + E_{i,\min }^{Windm} }}{2} \hfill \\ \end{gathered} \right.$$

When the filtering relative tolerance of $E_{i + 1}^{Windm}$ is equal to 0.2, or the Max Num Extrema of the remaining $E_{i + 1}^{Windm}$ is equal to 1, the EMD decomposition is completed. The wind power curve EMD decomposition concludes. The wind power curve equals the sum of the high and low-frequency components obtained through EMD decomposition:

5 $$E^{Windm} = \sum\nolimits_{i = 1}^{N} {IMF_{i}^{Windm} } + R^{Windm}$$

**Figure 4:**
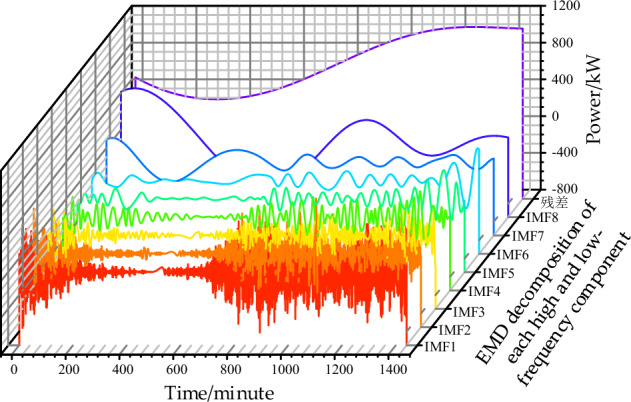
Wind power curve EMD decomposition of each order component curve.

Using formulas (4), (5) to perform EMD decomposition on the high-frequency wind power fluctuation curve in Fig. 1a, the obtained components of each order (IMF) are shown in Fig. 4 below:

In Fig. 4, it can be observed that through Empirical Mode Decomposition (EMD), the high-frequency strong fluctuation component and the low-frequency component of the wind power curve are obtained. The high-frequency component of the Intrinsic Mode Functions (IMF) exhibits a characteristic of basic symmetry in the envelope line. This mathematical model feature allows the positive and negative components to cancel each other out to a certain extent. On the hardware device level, this implies the need for an energy storage device capable of efficiently storing high-frequency fluctuations, and in this context, a flywheel energy storage system is chosen.

The flywheel energy storage system is selected as the energy storage and smoothing device for the high-frequency fluctuation component of wind power. The flywheel energy storage system can distribute the mechanical power of wind power when high-frequency positive components are expected and supplement the electrical power of wind power during high-frequency negative components. The simplification of the electrochemical energy storage process from mechanical-electric-chemical storage-discharge to mechanical-electric discharge enables the flywheel energy storage system to have higher energy storage efficiency. In addition, in the absence of unexpected failures, the flywheel energy storage system can undergo an infinite number of charge–discharge cycles throughout its lifecycle, minimizing its environmental impact.

**Figure 5:**
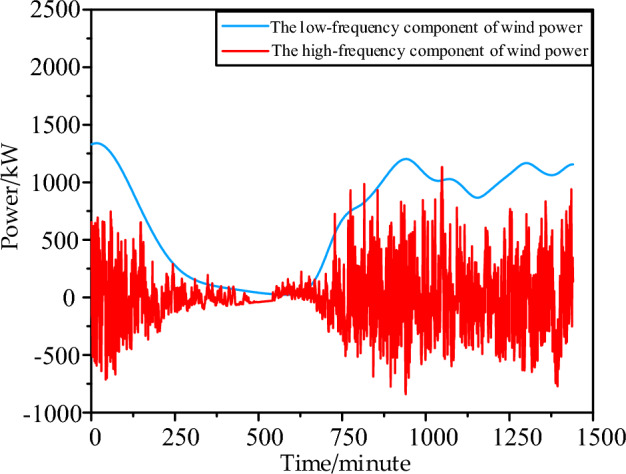
High and low-frequency reconfiguration of wind power after EMD decomposition.

Figure 5 shows the aggregated high-frequency wind power fluctuation curve obtained by combining IMF1-IMF7. It can be observed that positive and negative fluctuation quantities are still essentially complementary. However, the charging efficiency η_c_ and discharging efficiency η_d_ of the energy storage system are inevitably less than 1. This results in 1-ηcηd times of high-frequency components remaining after positive charging and negative discharging. In previous literature, a 0 baseline was often used in EMD decomposition for capacity configuration, leading to the persistence of large-scale negative fluctuations. Here, a capacity configuration method with a variable baseline is proposed, where the 0 baseline is lowered to increase the positively chargeable electricity and reduce the negatively compensatory electricity. This further promotes the smoothing of high-frequency power fluctuations. The model for flywheel energy storage capacity configuration based on EMD high-low frequency decomposition with a variable baseline can be formulated as follows:

6 $$\begin{gathered} W^{H} = \sum\nolimits_{t = 1}^{T} {P^{Wave} \cdot \left| {E_{t}^{Wave} } \right| \cdot \Delta t} + \sigma_{d} \cdot P^{FS} \cdot S^{FS} \hfill \\ s.t.\left\{ \begin{gathered} E_{t}^{WindHm} = \sum\nolimits_{i = 1}^{{N_{i} }} {IMF_{i,t}^{Windm} } \hfill \\ E_{t}^{WindLm} = \sum\nolimits_{{N_{i + 1} }}^{{N_{\max } }} {IMF_{i,t}^{Windm} } \hfill \\ E_{t}^{Wave} = E_{t}^{WindHm} - E_{t}^{FSC} + E_{t}^{FSD} - E^{Base} \hfill \\ E_{{{\text{i}},\min }}^{WindHm} \le E^{Base} \le E_{{{\text{i}},\max }}^{WindHm} \hfill \\ E_{t}^{WindLastm} = E_{t}^{WindLm} + E^{Base} \hfill \\ 0 \le E_{t}^{FSC} \le \alpha^{FSC} \cdot S^{FS} \cdot v_{t}^{FSC} \hfill \\ 0 \le E_{t}^{FSD} \le \alpha^{FSD} \cdot S^{FS} \cdot (1 - v_{t}^{FSC} ) \hfill \\ v_{t}^{FSC} \in \left\{ {0,1} \right\} \hfill \\ 0 \le S^{FS} \le S_{\max }^{FS} \hfill \\ \beta_{\min }^{FS} \cdot S^{FS} \le s^{FS} \le \beta_{\max }^{FS} \cdot S^{FS} \hfill \\ s_{t}^{FS} = s_{t - 1}^{FS} + \eta^{FSC} \cdot E_{t}^{FSC} - E_{t}^{FSD} /\eta^{FSD} \hfill \\ \delta_{SOC} (0) = \delta_{SOC} (24) \hfill \\ \end{gathered} \right. \hfill \\ \end{gathered}$$

In the formula, *W*^*H*^ is the Comprehensive cost for handling high-frequency wind power fluctuations. *σ*_*d*_ is the coefficient of daily cost for flywheel energy storage over the total lifecycle cost, *P*^*FS*^ is the investment cost of the flywheel energy storage unit per kWh, S^*FS*^ is the optimal energy storage capacity of the flywheel energy storage unit; $E_{t}^{Wave}$ is the small fluctuation component that remains after the suppression of high-frequency fluctuation, *P*^*Wave*^ is the penalty price coefficient for the remaining small fluctuation; Δt is the minimum scheduling hour; $E_{t}^{WindHm}$ and $E_{t}^{WindLm}$ are the accumulations of high-frequency and low-frequency fluctuation curves, respectively, after EMD decomposition with IMF_i as the boundary; $E_{t}^{WindLastm}$ is the total power accumulation of wind power after the suppression of high and low-frequency by flywheel energy storage; $E_{t}^{FSC}$ and $E_{t}^{FSD}$ are the power storage and release of flywheel energy storage; *E*^*Base*^ is the variable baseline power set to adapt to the charging power shortage caused by charging and discharging efficiency; $E_{i,\min }^{WindHm}$ and $E_{i,\max }^{WindHm}$ are the minimum negative power and maximum positive power of wind power after high-frequency reconstruction, respectively; *α*^*FSC*^ and *α*^*FSD*^ are the ratio coefficients of the upper limit power and capacity of flywheel energy storage unit for charging and discharging, respectively; $V_{t}^{FSC}$ is the energy storage status indicator position of the flywheel energy storage unit, with 1 indicating storage and 0 indicating release; $S_{\max }^{FS}$ is the configured capacity upper limit of the flywheel energy storage unit; $\beta_{{F_{\min } }}^{S}$ and $\beta_{{F_{\max } }}^{S}$ are the minimum and maximum storage energy ratios, respectively; $S_{t}^{FS}$ is the real-time energy storage energy; *η*^*FSC*^ and *η*^*FSD*^ are the energy storage and release efficiency of the flywheel energy storage unit, respectively; *δ*_*SOC*_ refers to the state of charge of the energy storage; *δ*_*SOC*_(0) indicates the state of charge at 0 o'clock; *δ*_*SOC*_(24) represents the state of charge at 24 o'clock.

**Figure 6:**
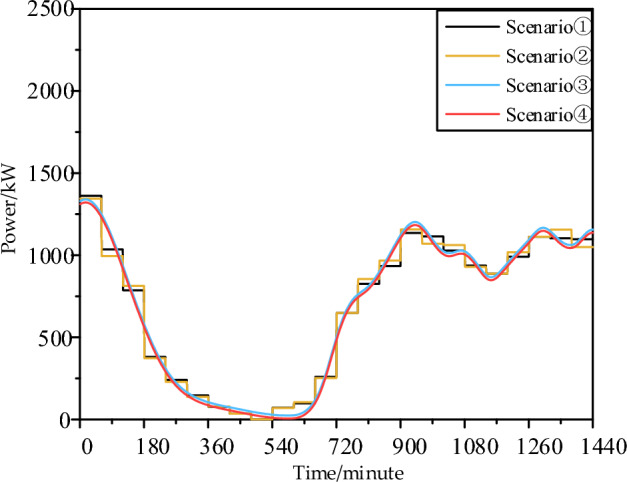
Wind power comparison between 1 min and one hour after high-frequency fluctuation levelling, Scenario ①: Wind power 60-min average power curve without random fluctuations. Scenario ②: Wind power 60-min average power curve with random fluctuations. Scenario ③: Low-frequency component of wind power 1-min power with random fluctuations. Scenario ④: The sum of the low-frequency power component and the high-frequency power component of wind power at 1 min, after being smoothed by flywheel energy storage.

On the basis of the wind power curve being decomposed and reconstructed with high and low frequencies using formulas (1–5) by EMD, the results of configuring flywheel energy storage to mitigate high-frequency fluctuations using formula (6) are as follows:

By comparing Fig. 3a,d and Fig. 6, it can be observed that the high-frequency flywheel energy storage configuration strategy in this section significantly reduces the differences in power generation between different time scales. This significantly improves the guidance of the same dispatch decision for sudden random power fluctuations at different time scales, enabling the achievement of a basic consistency in the maximum fluctuation range, fluctuation ratio, and fluctuation conservatism between the 1-min and 60-min two-stage robust dispatch based on robust set theory.

The 1-min time scale is too small, leading to a significant increase in computational complexity for the two-stage robust dispatch in the following sections. To address this issue, the average over 1 h, EWindLmh t, can be taken.

7 $$E_{t}^{{WindLm{\text{h}}}} = \frac{1}{60}\sum\nolimits_{60*t - 59}^{60*t} {E_{t}^{WindLm} }$$

### Configuration without uncertainty considerations

In the previous section, the flywheel energy storage was configured through EMD decomposition to achieve the unity of overall power generation from wind and energy storage at multiple time scales. Subsequently, subjecting the overall system to uncertain robust planning can result in a smaller fluctuation range and more meaningful planning decisions. The following analyses are based on the low-frequency results generated in previous sections.

However, the two-stage robust planning model considering uncertainty needs to be based on the linearized model of low-frequency energy storage capacity configuration for a data center microgrid without considering wind power uncertainty. Therefore, the foundational modeling of the data center microgrid planning is as follows:

#### Outer storage capacity configuration

8 $$\begin{gathered} W^{TZ} = \sigma_{d}^{XHL} \cdot L_{\max }^{XHL} \cdot L_{per}^{XHL} + \sigma_{d}^{Lss} \cdot S_{\max }^{Lss} \cdot S_{per}^{{L{\text{ss}}}} \hfill \\ s.t.\left\{ \begin{gathered} 0 \le L_{\max }^{XHL} \le L_{MAX}^{XHL} \hfill \\ 0 \le S_{\max }^{Lss} \le S_{MAX}^{Lss} \hfill \\ \sigma_{d}^{XHL} = \frac{1}{365}\frac{{\varphi (1 + \varphi )^{{Y_{XHL} }} }}{{(1 + \varphi )^{{Y_{XHL} }} - 1}} \hfill \\ \sigma_{d}^{Lss} = \frac{1}{365}\frac{{\varphi (1 + \varphi )^{{Y_{Lss} }} }}{{(1 + \varphi )^{{Y_{Lss} }} - 1}} \hfill \\ \end{gathered} \right. \hfill \\ \end{gathered}$$

In the equation: W^TZ^ represents the cost of equipment capacity configuration; $\sigma_{d}^{XHL}$ and $\sigma_{d}^{LSS}$ are the daily discount rates for the lithium bromide absorption chiller and the ice storage system; ${\text{L}}_{{{\text{max}}}}^{{{\text{XHL}}}}$ and ${\text{S}}_{{{\text{max}}}}^{{{\text{LSS}}}}$ are the configured power and capacity for the lithium bromide absorption chiller and ice storage system, respectively; ${\text{L}}_{{{\text{per}}}}^{{{\text{XHL}}}}$ and ${\text{S}}_{{{\text{per}}}}^{{{\text{Lss}}}}$ are the investment costs per unit capacity for the lithium bromide absorption chiller and ice storage system; φ is the annual discount rate, and Y_XHL_ and Y_LSS_ are the lifespans of the lithium bromide absorption chiller and ice storage system.

#### Inner data center microgrid

In the inner layer, the coordinated operation of the data center microgrid is modeled

9 $$W^{YX} = \sum\nolimits_{t = 1}^{T} {\left( {E_{t}^{buy} P_{t}^{{{\text{e}}buy}} - E_{t}^{sell} P_{t}^{esell} + E_{t}^{MT} P^{eMT} + E_{t}^{Windcur} P^{cur} + L_{t}^{XHL} P^{lxhl} + L_{t}^{{L{\text{ssC}}}} P^{lssc} } \right)}$$

In the equation: *W*^*YX*^ represents the comprehensive operating cost of the data center microgrid; $E_{t}^{buy}$ and $E_{t}^{sell}$ represent the power purchased and sold to the grid by the microgrid, respectively; $P_{t}^{ebuy}$ and $P_{t}^{esell}$ represent the purchase and sale prices of electricity to the grid by the microgrid, respectively; $E_{t}^{MT}$ is the power generated by the gas turbine, and *P*^*eMT*^ is the unit generation cost of the gas turbine; $E_{t}^{Windcur}$ is the curtailed wind power, and *P*^*cur*^ is the unit penalty for curtailed wind power; $L_{t}^{XHL}$ and *P*^*lxhl*^ are the cooling power and unit cooling cost of the lithium bromide absorption chiller; $L_{t}^{LssC}$ and *P*^*lssc*^ are the cooling power and maintenance cost of the chilled water storage tank.

For each component of the inner data center microgrid, we build a specific model to fit its unique characteristics. All models are defined as follows.

##### Data center loads

The data center load can be divided into interactive and batch-processing. Interactive loads require immediate processing while batch processing loads need to be completed within a specified time limit. Reference^23^ defines batch processing loads with a maximum delay of 2 h. Therefore, the time-limited shiftable characteristic of data center loads can be modeled as follows:

10 $$A = \left[ \begin{gathered} {1,0,0,0,0,0,0,0,0,0,0,0,0,0,0,0,0,0,0,0,0,0,1,1 } \hfill \\ {1,1,0,0,0,0,0,0,0,0,0,0,0,0,0,0,0,0,0,0,0,0,0,1} \hfill \\ {1,1,1,0,0,0,0,0,0,0,0,0,0,0,0,0,0,0,0,0,0,0,0,0} \hfill \\ {0,1,1,1,0,0,0,0,0,0,0,0,0,0,0,0,0,0,0,0,0,0,0,0} \hfill \\ {0,0,1,1,1,0,0,0,0,0,0,0,0,0,0,0,0,0,0,0,0,0,0,0} \hfill \\ {0,0,0,1,1,1,0,0,0,0,0,0,0,0,0,0,0,0,0,0,0,0,0,0} \hfill \\ {0,0,0,0,1,1,1,0,0,0,0,0,0,0,0,0,0,0,0,0,0,0,0,0} \hfill \\ {0,0,0,0,0,1,1,1,0,0,0,0,0,0,0,0,0,0,0,0,0,0,0,0} \hfill \\ {0,0,0,0,0,0,1,1,1,0,0,0,0,0,0,0,0,0,0,0,0,0,0,0} \hfill \\ {0,0,0,0,0,0,0,1,1,1,0,0,0,0,0,0,0,0,0,0,0,0,0,0} \hfill \\ {0,0,0,0,0,0,0,0,1,1,1,0,0,0,0,0,0,0,0,0,0,0,0,0} \hfill \\ {0,0,0,0,0,0,0,0,0,1,1,1,0,0,0,0,0,0,0,0,0,0,0,0} \hfill \\ {0,0,0,0,0,0,0,0,0,0,1,1,1,0,0,0,0,0,0,0,0,0,0,0} \hfill \\ {0,0,0,0,0,0,0,0,0,0,0,1,1,1,0,0,0,0,0,0,0,0,0,0} \hfill \\ {0,0,0,0,0,0,0,0,0,0,0,0,1,1,1,0,0,0,0,0,0,0,0,0} \hfill \\ {0,0,0,0,0,0,0,0,0,0,0,0,0,1,1,1,0,0,0,0,0,0,0,0} \hfill \\ {0,0,0,0,0,0,0,0,0,0,0,0,0,0,1,1,1,0,0,0,0,0,0,0} \hfill \\ {0,0,0,0,0,0,0,0,0,0,0,0,0,0,0,1,1,1,0,0,0,0,0,0} \hfill \\ {0,0,0,0,0,0,0,0,0,0,0,0,0,0,0,0,1,1,1,0,0,0,0,0} \hfill \\ {0,0,0,0,0,0,0,0,0,0,0,0,0,0,0,0,0,1,1,1,0,0,0,0} \hfill \\ {0,0,0,0,0,0,0,0,0,0,0,0,0,0,0,0,0,0,1,1,1,0,0,0} \hfill \\ {0,0,0,0,0,0,0,0,0,0,0,0,0,0,0,0,0,0,0,1,1,1,0,0} \hfill \\ {0,0,0,0,0,0,0,0,0,0,0,0,0,0,0,0,0,0,0,0,1,1,1,0} \hfill \\ {0,0,0,0,0,0,0,0,0,0,0,0,0,0,0,0,0,0,0,0,0,1,1,1} \hfill \\ \end{gathered} \right]$$

11 $$\left\{ \begin{gathered} E_{t}^{Data} = E_{t}^{DataJH} + E_{t}^{DataPLast} \hfill \\ 0 \le E_{i,t}^{DataPY} \le A.*E_{t}^{DataPOri} \hfill \\ E_{t}^{DataPOri} = \sum\nolimits_{{{\text{i}} = 1}}^{24} {E_{i,t}^{DataPY} } \hfill \\ E_{t}^{DataPLast} = \sum\nolimits_{j = 1}^{24} {E_{t,j}^{DataPY} } \hfill \\ \end{gathered} \right.$$

In the equation, *A* is the auxiliary matrix for modeling the time-limited and shiftable characteristics of data center loads, $E_{t}^{Data}$ is the total electrical load after demand response in the data center, $E_{t}^{DataJH}$ is the non-deferrable interactive load, $E_{t}^{DataLast}$ is the deferrable interactive load, $E_{i,t}^{DataPY}$ is the matrix of unordered batch processing load, and $E_{t}^{DataPOri}$ is the power matrix after limited-time transfer of batch processing load. The dimensions of $E_{i,t}^{DataPY}$ are the same as A, and the power at positions where A is 0 in $E_{i,t}^{DataPY}$ is also 0. The initial load of batch processing at time t can be allocated to rows from t to t + 2 in column t. Cumulatively adding the power matrix $E_{i,t}^{DataPY}$ after limited-time transfer column-wise yields the ordered power sequence of data center batch processing loads after demand response.

##### Load cooling system

The servers in the data center emit a large amount of heat during power consumption. Cooling is primarily achieved through a refrigeration system composed of a cooling tower, a chilled water circulation pump, a chiller unit, and a chilled water circulation pump. This method consumes a significant amount of electricity. We are considering configuring a gas turbine to utilize its waste heat for a bromine lithium absorption chiller and a chilled water tank. This aims to increase the energy efficiency of the gas turbine and reduce the overall operating costs of the data center cooling system. The power consumption for a cooling capacity of 2000 kW is approximately 10 kilowatts.

12 $$\left\{ \begin{gathered} L_{t}^{Data} = \alpha_{E2L}^{Data} \cdot E_{t}^{Data} \hfill \\ L_{t}^{Data} = L_{t}^{Water} + L_{t}^{XHLdir} + L_{t}^{LssD} \hfill \\ L_{t}^{Water} = \eta_{E2L}^{Water} \cdot E_{t}^{Water} \hfill \\ L_{t}^{XHL} = L_{t}^{XHLdir} + L_{t}^{LssC} \hfill \\ L_{t}^{XHL} = \alpha_{E2L}^{XHL} \cdot E_{t}^{XHL} \hfill \\ L_{t}^{XHL} \le \eta_{H2L}^{XHL} \cdot H_{t}^{MT} \hfill \\ H_{t}^{MT} = \alpha_{E2H}^{MT} \cdot E_{t}^{MT} \hfill \\ 0 \le E_{t}^{MT} \le E_{\max }^{MT} \hfill \\ 0 \le L_{t}^{Water} \le L_{\max }^{Water} \hfill \\ 0 \le L_{t}^{XHL} \le L_{\max }^{XHL} \hfill \\ \end{gathered} \right.$$

In the equation: $\alpha_{E2L}^{Data}$ is the coefficient of the ratio between the power consumption and cooling power of the data center load; $L_{t}^{Data}$ is the cooling power demand of the data center, including water circulation cooling ($L_{t}^{water}$), bromine lithium absorption chiller cooling power ($L_{t}^{XHLdir}$), and chilled water tank discharge cooling power ($L_{t}^{LssD}$); $\eta_{E2L}^{Data}$ and $E_{t}^{water}$ are the water cooling efficiency and power consumption, respectively; $\eta_{{X_{E2L} }}^{HL}$ is the efficiency of the bromine lithium absorption chiller; $L_{t}^{LssC}$ is the cooling power of the bromine lithium chilled tank; $\alpha_{E2L}^{XHL}$ is the coefficient of the ratio between the power consumption ($E_{t}^{XHL}$) and cooling power ($L_{t}^{XHL}$) of the bromine lithium absorption chiller; *αMT E2H* is the ratio coefficient between the power generation ($E_{t}^{MT}$) and heat production ($H_{t}^{MT}$) of the gas turbine; $E_{\max }^{MT}$, $L_{\max }^{Water}$, and $L_{\max }^{XHL}$ are the maximum power of the gas turbine, maximum power of water circulation cooling, and maximum cooling power of the bromine lithium absorption chiller, respectively.

The mathematical model for configuring the capacity of the ice storage system ($S_{\max }^{Lss}$) is as follows:

13 $$\left\{ \begin{gathered} S_{1}^{Lss} = S_{0}^{Lss} + \eta^{LssC} \cdot L_{1}^{LssC} - L_{1}^{LssD} /\eta^{LssD} \hfill \\ S_{{{\text{t + }}1}}^{Lss} = S_{t}^{Lss} + \eta^{LssC} \cdot L_{t}^{LssC} - L_{t}^{LssD} /\eta^{LssD} \hfill \\ 0 \le L_{t}^{LssC} \le \alpha^{LssC} \cdot S_{\max }^{Lss} \hfill \\ 0 \le L_{t}^{LssD} \le \alpha^{LssD} \cdot S_{\max }^{Lss} \hfill \\ S_{T}^{Lss} = S_{0}^{Lss} \hfill \\ \alpha_{L}^{Lss} \cdot S_{\max }^{Lss} \le S_{{\text{t}}}^{Lss} \le \alpha_{U}^{Lss} \cdot S_{\max }^{Lss} \hfill \\ \end{gathered} \right.$$

In the equation, $S_{MAX}^{Lss}$ is the upper limit of the ice storage capacity configuration; $S_{0}^{Lss}$ and $S_{T}^{LSS}$ satisfy the constraint of equal capacity at the initial and final time; *η*^*LssC*^ and *η*^*LssD*^ are the storage and release efficiency of the ice storage system, respectively; $L_{t}^{LssC}$ and $L_{t}^{LssD}$ are the storage and release cooling power, respectively; *α*^*LssC*^ and *α*^*LssD*^ are the ratios of the maximum storage and release power of the ice storage system to the capacity upper limit, respectively; $\alpha_{L}^{Lss}$ and $\alpha_{U}^{Lss}$ are the lower and upper limits of the capacity of the ice storage system, respectively. For the convenience of the subsequent KKT processing, the cooling storage cost is added to the objective function in Eq. (8), thereby avoiding the introduction of binary variables for conflicting constraints between cooling storage and release states.

##### Wind power and load constraints

14 $$\left\{ \begin{gathered} 0 \le E_{t}^{Wind} \le E_{t}^{WindLmh} \hfill \\ 0 \le E_{t}^{Windcur} \le E_{t}^{WindLmh} \hfill \\ E_{t}^{Wind} + E_{t}^{Windcur} = E_{t}^{WindLmh} \hfill \\ \end{gathered} \right.$$

The equation is: $E_{t}^{WindLmh}$, $E_{t}^{Wind}$, and $E_{t}^{Windcur}$ represent the low-frequency component, scheduled output, and curtailed power of the wind power forecast, respectively.

##### External grid interaction constraints

15 $$\left\{ \begin{gathered} 0 \le E_{t}^{buy} \le E_{\max }^{buy} \hfill \\ 0 \le E_{t}^{sell} \le E_{\max }^{sell} \hfill \\ \end{gathered} \right.$$

The formula is as follows:

$E_{\max }^{buy}$ and $E_{\max }^{sell}$ represent the power limits for buying and selling electricity from/to the grid in the data center microgrid. For the convenience of subsequent KKT processing, different unit costs for buying and selling electricity are incorporated within the objective function (Eq. 8), thereby avoiding the introduction of binary variables to constrain conflicting states of buying and selling electricity. The system power balance constraints are presented as follows:

16 $$E_{t}^{buy} + E_{t}^{Wind} + E_{t}^{MT} = E_{t}^{sell} + E_{t}^{XHL} + E_{t}^{Data} + E_{t}^{Water}$$

### Configuration with wind power box uncertainty set

On the basis of the data center microgrid planning model, without considering wind power uncertainty in the previous section, it is only necessary to replace the constant power values of wind power and data center loads with uncertainty curves constrained by the fluctuation range and total fluctuation of the box uncertainty set. By utilizing the improved C&CG and KKT algorithms for iterative solving, the optimal capacity configuration considering system robustness can be obtained.

All configurations in this section, considering the wind power box uncertainty set, apply the two-stage robust method.

The two-stage robust planning problem is a min–max-min problem. The complexity of the model lies in handling the impact of stochastic low-frequency power $\hat{E}_{t}^{WindLm}$, stochastic data center interactive load $\hat{E}_{t}^{WindJH}$, and stochastic data center batch processing limited-time shift load initial power $\hat{E}_{t}^{DataPOri}$ on the scheduling and planning objective functions. Additionally, the nonlinear model is optimized through the iterative convergence of C&CG and KKT.

17 $$\begin{gathered} \mathop {\min }\limits_{{L_{\max }^{XHL} ,S_{\max }^{{L{\text{ss}}}} }} W^{TZ} + \mathop {\max }\limits_{{\widehat{E}_{t}^{WindLm} ,\widehat{E}_{t}^{DataJH} ,\widehat{E}_{t}^{DataPOri} }} \mathop {\min }\limits_{x} W^{YX} \hfill \\ s.t.\left\{ \begin{gathered} - D_{\max }^{{M_{i} }} \le \frac{{E_{t}^{{M_{i} }} - \widehat{E}_{t}^{{M_{i} }} }}{{E_{t}^{{M_{i} }} }} \le D_{\max }^{{M_{i} }} \hfill \\ T\sum\nolimits_{t = 1}^{T} {\frac{{\left| {E_{t}^{{M_{i} }} - \widehat{E}_{t}^{{M_{i} }} } \right|}}{{D_{\max }^{{M_{i} }} \cdot E_{t}^{{M_{i} }} }}} \le \Gamma_{\max }^{{M_{i} }} \hfill \\ h_{1} (y) = 0 \hfill \\ g_{1} (y) \le 0 \hfill \\ \begin{array}{*{20}c} {h_{2} (x) = 0} & { \to \lambda_{2} } \\ \end{array} \hfill \\ \begin{array}{*{20}c} {g_{2} (x) \le 0} & { \to \mu_{2} ,v_{2} } \\ \end{array} \hfill \\ \end{gathered} \right. \hfill \\ \end{gathered}$$

In the formula: M^i^ represents the ith random variable name, $E_{t}^{{M^{i} }}$ and $\hat{E}_{t}^{{M^{i} }}$ are the forecast power and stochastic power, respectively; $DM_{max}^{{M^{i} }}$ and $\Gamma{M}_{max}^{{M^{i} }}$ are the fluctuation range and conservatism of the box uncertainty set, respectively; *y* is the first-stage decision variable, corresponding to the outer capacity configuration variable in formula (8); h_1_(y) and g_1_(y) are the equality and inequality constraints of the first stage, respectively; h_2_ (x), g_2_ (x), λ_2_, and μ_2_ are the parameters of the second-stage minimum scheduling cost, corresponding to the formulas (9–16) in the previous text, preparing for the KKT condition handling of the max–min nonlinear problem in the following text.

#### The first-stage: C&CG algorithm

The objective of the first-stage master problem is to make decisions on the optimal equipment configuration capacities, $L_{{\max }}^{{XHL}}$ and $S_{{\max }}^{{Lss}}$. The input to the model consists of worst-case scenario constant curves for wind power and load, denoted as ${E_{{Bad,t}}^{{WindLm}} }$, ${E_{{Bad,t}}^{{DataJH}} }$, and ${E_{{Bad,t}}^{{DataPOri}} }$, respectively, instead of random variables. The objective function minimizes the total investment cost, W^TZ^, and the sum of the comprehensive operational cost cut planes, denoted as *η*, subject to the constraints imposed by the C&CG algorithm.

18 $$\begin{gathered} \begin{array}{*{20}c} {\mathop {\min }\limits_{{L_{\max }^{XHL} ,S_{\max }^{{L{\text{ss}}}} }} } & {W_{iter}^{TZ} + \eta \left( {E_{Bad,t}^{WindLm} ,E_{Bad,t}^{DataJH} ,E_{Bad,t}^{DataPOri} } \right)} \\ \end{array} \hfill \\ s.t.\left\{ \begin{gathered} H_{1} (Y) = 0 \hfill \\ G_{1} (Y) \le 0 \hfill \\ H_{2} (X) = 0 \hfill \\ G_{2} (X) \le 0 \hfill \\ \eta \ge W_{iter}^{YX} ,\forall E_{t,iter}^{{M_{i} }} \in E_{BAD}^{{M_{i} }} \hfill \\ UB_{iter} = W_{iter}^{TZ} + \eta \hfill \\ \end{gathered} \right. \hfill \\ \end{gathered}$$

The Column-and-Cut Generation (C&CG) algorithm is mainly reflected in the fact that with each iteration, a set of worst-case source-load scenario curves is added, causing the constraint dimension of the base model to expand continuously. It's important to note that the dimensions of the variables for fixed equipment and operating powers of the configured equipment continuously expand to ITER × 24, while the dimensions of the newly configured equipment capacity variables always remain 1 × 1. The model's C&CG cut plane variable, *η,* is consistently greater than the value of the second-stage operational cost objective function, W^YX^, under all worst-case source-load scenarios. Since the worst-case scenario set is finite, the value of the cut plane variable η for the finite scenario set must be less than the optimal maximum value of the second-stage max–min problem. Therefore, the result obtained from solving the first-stage master problem is a convergent lower bound, *LB*_*iter*_.

#### The second stage: Karush–Kuhn–Tucker (KKT) conditions

The input of the second-stage sub-problem is the equipment capacity configuration results (*LXHL max* and *SLss max*) obtained from the first stage. The decision variables are the worst-case wind power (*EWindLm Bad,t*), worst-case data center interaction load (*EDataJH Bad,t*), and worst-case data center batch processing initial load (*EDataPOri Bad,t*), satisfying the fluctuation range and conservatism conditions. The objective function is to minimize the overall operating cost (*W*^*YX*^). The original two-level max–min *W*^*YX*^ nonlinear model is transformed into a single-level min *W*^*YX*^ using the KKT conditions.

19 $$\begin{gathered} \mathop { - \min }\limits_{{\widehat{E}_{t}^{WindLm} ,\widehat{E}_{t}^{DataJH} ,\widehat{E}_{t}^{DataPOri} }} W_{iter}^{YX} \left( {L_{\max }^{XHL} ,S_{\max }^{Lss} } \right) \hfill \\ s.t.\left\{ \begin{gathered} - D_{\max }^{{M_{i} }} \le \frac{{E_{t}^{{M_{i} }} - \widehat{E}_{t}^{{M_{i} }} }}{{E_{t}^{{M_{i} }} }} \le D_{\max }^{{M_{i} }} \hfill \\ T\sum\nolimits_{t = 1}^{T} {\frac{{\left| {E_{t}^{{M_{i} }} - \widehat{E}_{t}^{{M_{i} }} } \right|}}{{D_{\max }^{{M_{i} }} \cdot E_{t}^{{M_{i} }} }}} \le \Gamma_{\max }^{{M_{i} }} \hfill \\ L = W^{YX} + \lambda_{2} h_{2} (x) + \mu_{2} g_{2} (x) \hfill \\ \frac{\partial L}{{\partial x}} = 0 \hfill \\ \lambda_{2} \ne 0 \hfill \\ 0 \le u_{2} \le (1 - v_{2} ) \cdot M \hfill \\ - v_{2} \cdot M \le g_{2} (x) \le 0 \hfill \\ h_{2} (x) = 0 \hfill \\ LB_{iter} = W_{iter}^{TZ} + W_{iter}^{YX} \hfill \\ \end{gathered} \right. \hfill \\ \end{gathered}$$

In the equation, L represents the augmented Lagrangian polynomial. The KKT conditions mainly include seven sets of constraints: original equality constraints, original inequality constraints, equality constraints from L on the max–min inner decision variables (*∂L/∂x*), constraints where the Lagrange multiplier λ_2_ for the equality is not equal to 0, constraints where the Lagrange multiplier μ_2_ for the inequality is greater than or equal to 0, and two sets of decoupling constraints using binary variables v_2_ combined with the Big-M method to ensure the product of μ_2_g_2_ (x) is equal to 0. When solving the second-stage sub-problem, since the values of the capacity configuration Y are finite, the investment cost under the finite set must be biased towards larger values. Therefore, the result of solving the second-stage sub-problem is the convergence upper limit *UB*_*iter*_.

#### Integration: iterative convergence conditions

Based on the upper and bottom bounds in the previous two stages, we now employ the iterative convergence conditions to achieve the optimal configuration of energy storage capacity in the face of the wind power box uncertainty set.

As the number of worst-case scenarios in the uncertainty set increases, it becomes increasingly close to the most adverse scenario. Therefore, with an increasing number of iterations, the lower bound (*LB*_*iter*_) obtained from solving the first-stage C&CG problem in Eq. (18) tends to approach the upper bound (*UB*_*iter*_) obtained from solving the second-stage KKT problem in Eq. (18). It is only necessary to set a proportionality threshold, denoted as *θ*, to determine the desired level of closeness between the two. Once this condition is satisfied, a robust optimal planning result that meets the required solution accuracy is achieved.

20 $$\frac{{UB_{iter} - LB_{iter} }}{{UB_{iter} }} \le \theta$$

## Results

In the case of a certain data center microgrid in Jilin Province, the architecture is illustrated in Fig. 1. The gas turbine capacity is 600 kW, the wind turbine capacity is 1500 kW, the data center load is 2000 kW, and the power limitation for grid interaction by the transformer is 1000 kW. The power consumption for water circulation cooling is 7 kW. The stochastic source-load curve for a 1-min time scale is shown in Appendix Fig. S1, and the peak-valley electricity prices are provided in Table A1. The coefficients for the mathematical model are given in Table A2. The programming environment used is MATLAB 2018B, along with Gurobi 9.5.2.

### EMD-based configuration of flywheel energy storage

When implementing the case study, the first step involves configuring flywheel energy storage based on EMD decomposition to suppress high-frequency fluctuations in wind power. This facilitates a closer alignment of source-load curves across multiple time scales, enhancing the general guidance of scheduling decisions at different time scales and, consequently, improving system stability.

**Figure 7:**
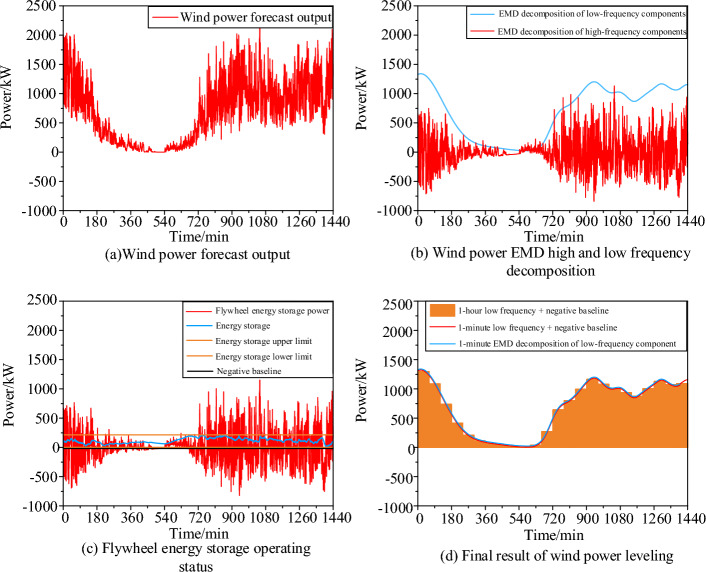
Smoothing results of high-frequency fluctuations in wind power.

**Figure 8:**
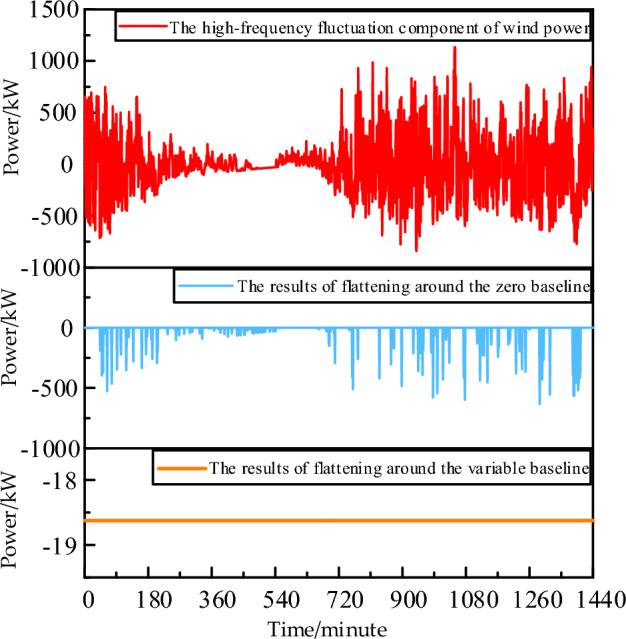
Comparison of the smoothing effect of zero datum line and variable datum line.

As shown in Fig. 7, Fig. 7a presents a wind power forecast output considering random fluctuations, which is very rough. Such strong power fluctuations can cause drastic scheduling changes in the interaction power between the gas turbine and the grid in the data center microgrid, adversely affecting the system's stability. Therefore, the paper adopts the capacity configuration based on the EMD decomposition algorithm, as shown in formulas (1–6), to stabilize the strong wind power fluctuations. First, Fig. 7b shows the result of reconstructing the high and low-frequency fluctuation components after EMD decomposition. The low-frequency component follows the trend of the original wind power curve, while the high-frequency component has the characteristics of symmetric amplitude fluctuation about the positive and negative half cycles. This provides extremely favorable conditions for minimizing the capacity configuration of high-frequency and high-efficiency energy storage devices. Based on these results, a flywheel energy storage device with an efficiency of over 0.9 is selected, and frequent 1-min alternating charging and discharging activities are carried out to neutralize the positive and negative half-axis symmetrical fluctuation components into a straight line. Considering that the charging and discharging efficiency is less than 1 if 0 kW is still used as the baseline, the smoothing result will be as shown in Fig. 8.

The strategy of the zero baseline results in the negative power retaining 1-ηC ηD times the high-frequency component. On the other hand, the variable baseline strategy involves lowering the zero baseline, increasing the rechargeable positive power, and decreasing the compensating power for the negative side, thus further suppressing high-frequency power fluctuations into a straight line. It's worth noting that if there are extremely large amplitude components in both positive and negative directions, some points may still protrude on the flattened negative baseline. The protruding positive fluctuations can be eliminated through curtailment, and the flattened negative side can be achieved through price-responsive demand.

**Figure 9:**
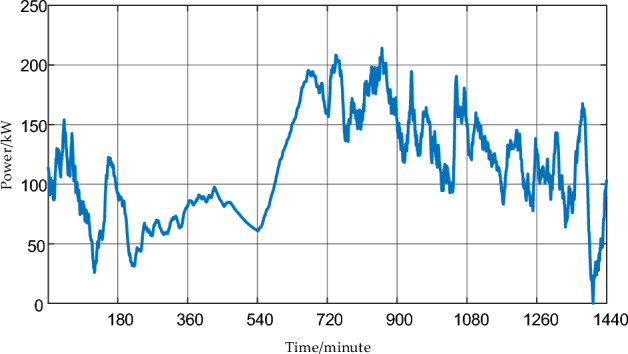
The state of charge (SOC) variation of the flywheel energy storage.

Figure 7c illustrates the operational state of the flywheel energy storage under the variable baseline mode, where the power change amplitude is large, but the capacity change amplitude is small. The power change trend is similar to the high-frequency component power trend of wind power, while the capacity change trend reflects the asymmetrical electrical characteristics between positive and negative high-frequency components. Figure 7d shows the negative baseline obtained after flattening the high-frequency component with the flywheel energy storage and its cumulative addition with the low-frequency component of wind power. The curve has a stable and coherent amplitude within a fluctuation range of 20% and a fluctuation conservatism of 50%. The variation in the state of charge (SOC) of the flywheel energy storage is illustrated in Fig. 9. From the graph, it can be observed that the rapid response of the flywheel energy storage system has shown remarkable effectiveness in smoothing the high-frequency component of wind power.In this scenario, the capacity of the high-frequency flywheel energy storage device configured by EMD is 172.4747 kWh. Subsequently, by averaging the stable wind power curve at the 1-min timescale, a 1-h wind power curve suitable for the two-stage robust scheduling and planning is obtained. The variable count is reduced from 1440 to 24, significantly reducing computation time and optimizing convergence errors.

### Capacity configuration with uncertainty considerations

With the smoothed wind power curve achieved through smoothing and dimensionality reduction, a two-stage robust planning is conducted to configure the capacities of bromine lithium refrigeration machines and cold storage tanks that promote waste heat recovery. This is done to enhance the system's robust economic performance.

**Figure 10:**
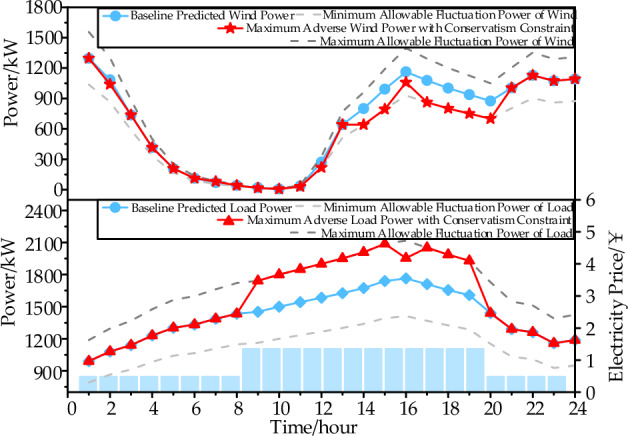
Worst Wind Power and Data Center Loads.

In the scenario with a fluctuation range of 20% and a conservative degree of 50% in the box uncertainty set, a two-stage robust planning analysis was conducted for the data center microgrid. The results are shown in Fig. 10. During peak hours with high electricity prices, the most adverse load power significantly increased, while the most adverse wind power decreased noticeably. This led to an increase in the power consumption from the gas turbine and electricity purchase from the grid, resulting in an overall increase in the system's operational cost.

**Figure 11:**
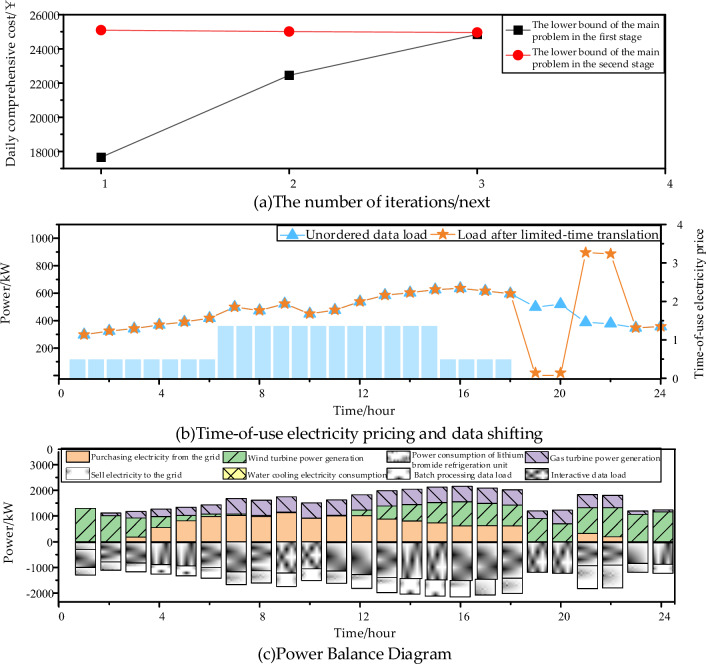
Analysis of two-stage robust planning algorithms.

The results shown in Fig. 11 further validate the convergence performance, limited-time shift ability of batch processing loads, and the characteristics of the system's electrical and cooling power output in the two-stage robust planning scenario for the data center microgrid., as observed in the figure, the two-stage robust planning converges within only three iterations, reducing the gap between the upper and lower bounds to within 1%. The economic benefits resulting from the decrease in unit electricity cost from the peak-hour price of 1.35 ￥/kWh to the off-peak-hour price of 0.48 ￥/kWh have a significant impact. The batch processing loads, which can be shifted for a limited time of 2 h, exhibit a noticeable decrease in power during the 19:00–20:59 period and a significant increase during the 21:00–22:59 period.

**Figure 12:**
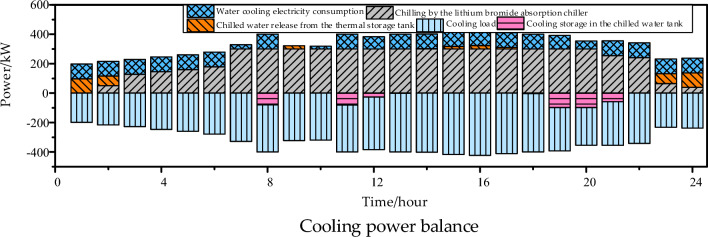
Cooling power balance.

From Figs. 11 and 12, it can be observed that during off-peak electricity pricing periods when the gas turbine is not in operation, the chilled water tank releases the stored cooling energy from daytime. The operating hours of the lithium bromide chiller coincide with the gas turbine startup period. The proportion of cooling power recovered from waste heat is relatively high, contributing to significant energy savings and environmental benefits. At this time, the configured chilled water tank capacity is 689.68 kWh, and the configured lithium bromide chiller capacity is 300 kW.

## Discussion

### Conservative degree and uncertainty fluctuations

In order to further explore the impact of different uncertainties in wind power and load on the proposed strategies in this study, this section conducts a case analysis on the key parameters of uncertainty range and conservatism in the commonly used box uncertainty set model. On the one hand, the effects of uncertainty range and conservatism parameters on the results of EMD and two-stage robust planning are displayed through three-dimensional diagrams. On the other hand, this analysis aims to derive optimal configuration solutions for a specific uncertain parameter scenario in a wind power microgrid.

**Figure 13:**
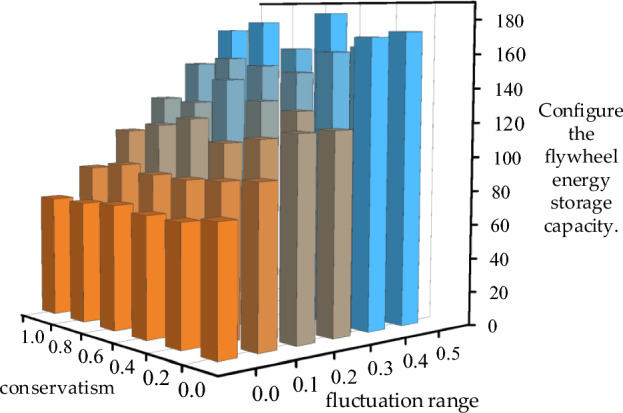
Impact of wind power uncertainty parameters on high-frequency energy storage configurations.

Continuing with the wind power curve shown in Fig. 3a, which already has strong fluctuations, introducing uniform distribution uncertainty components within the discrete fluctuation range and fluctuation conservatism parameters results in a new wind power curve with even greater fluctuations. The EMD decomposition for configuring flywheel energy storage capacity is shown in Fig. 13: the optimal configuration of flywheel energy storage capacity is strongly and positively correlated with the wind power fluctuation range and weakly correlated with the wind power fluctuation conservatism, i.e., the maximum allowable total wind power fluctuation.

**Figure 14:**
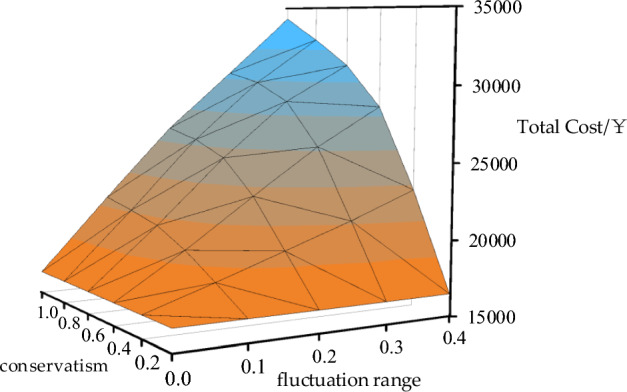
Impact of wind power uncertainty parameters on the total cost of robust planning.

In the context illustrated in Fig. 6, the 1-min highly fluctuating wind power curve is smoothed using EMD to obtain a 1-h smooth wind power curve. Subsequently, discrete fluctuation range and fluctuation conservatism parameters are introduced to limit the range. Within these constraints, the most unfavorable wind power load curves are determined, and the optimal investment and operating costs for the worst-case scenario are considered. The resulting relationship between conservatism, fluctuation range, and comprehensive costs is depicted in Fig. 14. When the fluctuation range is 0 or the fluctuation conservatism parameter is 0, it is observed that the optimal configuration results in the same value. This is because either of these parameters being 0 implies that the wind power and load curves are the original curves. The three-dimensional surface exhibits a double upward slope, indicating that as the maximum allowable fluctuation or range increases, the system's comprehensive investment and operating costs monotonically increase.

Finally, by analyzing the fluctuation of actual wind power and load data in a specific region over recent years, the most appropriate fluctuation range and conservatism parameters are determined. Subsequently, points are identified within the three-dimensional stereogram shown in Figs. 11 and 12 to obtain the optimal capacity configuration results.

### Robustness checks: coupled time scale parameters

In the study, 1 min is chosen as the minimum time interval to simulate real-time wind power curves. After smoothing the 1-min curve using EMD as shown in Fig. 6, the time dimension is expanded by 60 times to a 1-h interval, simulating the wind power curve for day-ahead scheduling. Subsequently, a two-stage robust planning is conducted. To illustrate the significance of this data processing, case analysis is performed for different intervals of coupled time scale parameters, and the calculated time and error rate are presented in Table 2 below:

**Table 2:** Characteristic parameters of wind power box fluctuation models at multiple time scales.

Coupled time scale parameters	1 min	5 min	15 min	60 min
Robust planning computation time/s	82,038	5114	272	19
Percentage of Time Consumption	1	6.23%	0.33%	0.02%
Error Rate	0	− 4.25%	− 4.81%	− 5.17%

After smoothing the large fluctuations in wind power to a smooth curve using EMD, the differences in wind power output power between multiple time scales become minimal. The two-stage robust planning based on the KKT and C&CG algorithms in the MATLAB + Gurobi environment is a first-order mixed-integer linearization problem. The dimension of the variables for one day at a 1-min time scale is 1440, while at a 1-h time scale, the dimension is 24. This reduction in the dimension of the variables for the linear programming problem AX = B by 60 times, under the same 1% convergence error, reduces the calculation time from 82,038 s to 19 s. When the time interval is expanded to 1 h, the amplitude fluctuations of wind power are further smoothed, resulting in a reduction in costs such as main grid purchasing and wind curtailment, leading to a -5.17% error. In summary, with the expansion of the coupling time scale from 1 min to 1 h, the calculation time is shortened to 0.02%, and the error rate is only − 5.17%, confirming that the proposed strategy is efficient and reliable.

## Conclusion

In addressing the challenges of strong fluctuations and large variability in the power output of small-capacity wind turbines, the paper initially employs the Empirical Mode Decomposition (EMD) algorithm, which demonstrates significant advantages in nonlinear high-low frequency decomposition. The algorithm is used to decompose and reconstruct the wind power fluctuations, particularly focusing on the 1-min nonlinear and highly fluctuating wind power curve.

The paper proposes a hybrid energy storage configuration strategy suitable for microgrids with small-capacity wind turbines, aiming to suppress strong wind power fluctuations and enhance economic efficiency. Through EMD decomposition, the wind power signal is separated into high-frequency and low-frequency components. The high-frequency wind power signal is mitigated through flywheel energy storage, while the low-frequency wind power signal is subjected to the subsequent two-stage robust calculation for the data center. This process determines the optimal capacity configuration for the lithium bromide absorption chiller and the ice storage tank, ultimately achieving the economic optimization of the data center. This paper overcomes the challenges of the two-stage robust approach in dealing with scheduling issues at minute and hour levels, realizing a multi-timescale scheduling strategy. The strategy is accompanied by a detailed problem analysis, mathematical modeling, and case demonstrations, with the following conclusions:

1. The EMD decomposition algorithm effectively separates the high and low-frequency components of the 1-min nonlinear and strongly fluctuating wind power curve. The reconstructed high-frequency fluctuations exhibit characteristics of symmetry around the positive and negative axes. The proposed variable baseline flywheel energy storage capacity configuration model successfully suppresses large-range high-frequency fluctuations, resulting in a negative power line, effectively superimposed onto the low-frequency reconstructed wind power component.
2. The coupling of the high-frequency EMD stability suppression model with the low-frequency robust economic improvement model involves simplifying the 1440-dimensional 1-min smooth wind power curve to a 24-dimensional 1-h curve. This significant dimensionality reduction leads to a shortened computation time of 0.02% and an error rate of only − 5.17%, making the approach highly efficient and reliable.
3. Modeling of the novel data center microgrid with wind power is presented, focusing on variables related to limited-time shifting. The C&CG algorithm is employed to further expand the mathematical model to iter × 24 × 24 dimensions. Procedures involving KKT and partial derivatives of 24 × 24 matrix variables showcase a certain level of model innovation.
4. An analysis of the box uncertainty set, including fluctuation range and total fluctuation conservatism, is conducted. Three-dimensional gradient pattern graphs illustrate a strong positive correlation between the optimal flywheel energy storage capacity and fluctuation range limitation parameters. Systemwide comprehensive investment and operating costs exhibit positive correlations with fluctuation and fluctuation range.

### Supplementary Information

Supplementary Tables.

Supplementary Figure S1.

## Data Availability

Data available on request from the authors. The data that support the findings of this study are available from the corresponding author, upon reasonable request.
