# Supplementary material for: Hybrid energy storage configuration method for wind power microgrid based on EMD decomposition and two-stage robust approach
Source: Sci Rep. 2024 Feb 1;14:2733. doi: 10.1038/s41598-024-53101-4 (PMC10834578; doi:10.1038/s41598-024-53101-4)
Supplement: Supplementary file 1 — Supplementary Tables. [file 41598_2024_53101_MOESM1_ESM.docx]

Appendix

**Table 1.** Time of use electricity price of power grid

| **Time** | **Peak and Valley Periods** | **Electricity Price** |
| --- | --- | --- |
| 0:00-7:59 | Valley Periods | 0.48 |
| 8:00-20:59 | Peak Periods | 1.35 |
| 21:00-23:59 | Valley Periods | 0.48 |

**Table 2.** Example device parameters

| **Parameter Name** | **Value** | **Parameter Name** | **Value** |
| --- | --- | --- | --- |
| $D_{max}^{EWindm}$ | 0.3 | $P^{cur}$ | 0.5 |
| $\Gamma$ | 12/24 | $P^{lxhl}$ | 0.1 |
| $P^{Wave}$ | 0.75 CNY/kWh | $P^{lssc}$ | 0.0005 |
| $\sigma_{d}$ | 2.64*10^-4^ | $\alpha_{E2L}^{Data}$ | 0.2 |
| $P^{FS}$ | 3600 | $\eta_{E2L}^{Water}$ | 3.33 |
| $\alpha^{FSC}$ | 60 | $\alpha_{E2L}^{XHL}$ | 0.016 |
| $\alpha^{FSD}$ | 60 | $\eta_{H2L}^{XHL}$ | 0.25 |
| $\beta_{min}^{FS}$ | 0 | $\alpha_{E2H}^{MT}$ | 2 |
| $\beta_{max}^{FS}$ | 1 | $\eta^{LssC}$ | 0.95 |
| $\eta^{FSC}$ | 0.95 | $\eta^{LssC}$ | 0.95 |
| $\eta^{FSD}$ | 0.95 | $\alpha^{LssC}$ | 0.2 |
| $\varphi$ | 0.05 | $\alpha^{LssD}$ | 0.2 |
| $Y_{XHL}$ | 20 | $\alpha_{L}^{Lss}$ | 0.1 |
| $Y_{Lss}$ | 20 | $\alpha_{U}^{Lss}$ | 0.9 |
| $P^{eMT}$ | 0.732 | $\theta$ | 0.01 |
