# Supplementary material for: Hybrid energy storage configuration method for wind power microgrid based on EMD decomposition and two-stage robust approach
Source: Sci Rep. 2024 Feb 1;14:2733. doi: 10.1038/s41598-024-53101-4 (PMC10834578; doi:10.1038/s41598-024-53101-4)
Supplement: Supplementary file 2 — Supplementary Figure S1. [file 41598_2024_53101_MOESM2_ESM.docx]

**Appendix Figure**:S1
